# Revisiting the role of phospholipases C in virulence and the lifecycle of *Mycobacterium tuberculosis*

**DOI:** 10.1038/srep16918

**Published:** 2015-11-25

**Authors:** Fabien Le Chevalier, Alessandro Cascioferro, Wafa Frigui, Alexandre Pawlik, Eva C. Boritsch, Daria Bottai, Laleh Majlessi, Jean Louis Herrmann, Roland Brosch

**Affiliations:** 1 Institut Pasteur, Unit for Integrated Mycobacterial Pathogenomics, F-75015, Paris, France; 2 Université Paris Diderot, Sorbonne Paris Cité, Cellule Pasteur, Paris, France; 3 Dipartimento di Ricerca Traslazionale e delle Nuove Tecnologie in Medicina e Chirurgia, University of Pisa, Italy; 4 INSERM U1173, UFR Sciences de la Santé Simone Veil, Université Versailles-Saint-Quentin, 78180 Saint-Quentin en Yvelines, France; 5 Service de Microbiologie, Hôpital Raymond Poincaré, Assistance Publique Hôpitaux de Paris, 92380 Garches, France

## Abstract

*Mycobacterium tuberculosis*, the agent of human tuberculosis has developed different virulence mechanisms and virulence-associated tools during its evolution to survive and multiply inside the host. Based on previous reports and by analogy with other bacteria, phospholipases C (PLC) of *M. tuberculosis* were thought to be among these tools. To get deeper insights into the function of PLCs, we investigated their putative involvement in the intracellular lifestyle of *M. tuberculosis*, with emphasis on phagosomal rupture and virulence, thereby re-visiting a research theme of longstanding interest. Through the construction and use of an *M. tuberculosis* H37Rv PLC-null mutant (ΔPLC) and control strains, we found that PLCs of *M. tuberculosis* were not required for induction of phagosomal rupture and only showed marginal, if any, impact on virulence of *M. tuberculosis* in the cellular and mouse infection models used in this study. In contrast, we found that PLC-encoding genes were strongly upregulated under phosphate starvation and that PLC-proficient *M. tuberculosis* strains survived better than ΔPLC mutants under conditions where phosphatidylcholine served as sole phosphate source, opening new perspectives for studies on the role of PLCs in the lifecycle of *M. tuberculosis*.

Most of the ~130 mycobacterial species^1^ are harmless to humans, whereas a few pose major threats to human health and life. Among the latter is *Mycobacterium tuberculosis*, the etiological agent of tuberculosis, which transmits efficiently among humans and globally accounts for 9 million new tuberculosis cases and 1.5 million deaths each year^2^. Many factors have been reported that contribute to the outstanding efficacy of *M. tuberculosis* to infect its host and circumvent eradication by the immune system^3^,^4^. Genome-based studies and advanced gene knock-out techniques have been instrumental for the identification of numerous virulence factors of *M. tuberculosis* that seem to be important for its lifestyle as key pathogen^5^,^6^. Comparative sequence analyses were also important for finding polymorphisms useful for molecular epidemiology^7^ and evolutionary studies^8^. Among the different approaches, genomic comparison of environmental, saprophytic mycobacteria with clinically relevant mycobacteria may provide important information.

One of the potential differences emerging from the comparison of non-pathogenic with pathogenic mycobacterial species is the presence of genes encoding phospholipase C (PLC) in the latter. For example, *Mycobacterium abscessus*, which represents an exceptional, emerging pathogen within the large group of otherwise mostly harmless fast-growing mycobacteria^9^,^10^,^11^,^12^, encodes a PLC involved in the intracellular survival of *M. abscessus* in amoebae^13^. Moreover, PLC-encoding genes are also present in several species of the slow-growing mycobacteria, which constitute a subgroup in the 16 S rDNA-based mycobacterial phylogeny^14^ and harbour the great majority of mycobacterial pathogens. Only few studies have addressed the impact of PLC on virulence of these pathogens. The most well known of these studies targeted PLCs of a clinical *M. tuberculosis* strain (MT103) and reported that PLC-knock-out mutants of this strain were attenuated at later stages of infection^15^. Together with reported cytotoxic effects of PLC^16^, these results were taken up by numerous review articles on mycobacterial pathogenicity^5^,^17^,^18^,^19^, leading to the widespread supposition that PLCs were important virulence factors of *M. tuberculosis.*

In the present study, we thus sought to gain deeper insights into the molecular mechanisms by which PLCs might contribute to virulence of *M. tuberculosis*. PLCs from selected species of other bacterial genera, such as *Listeria monocytogenes* or *Clostridium perfringens*, are known to play a significant role in helping the bacteria to escape from phagosomal containment inside host cells by acting together with pore-forming proteins such as listeriolysin or perfringolysin^20^,^21^,^22^.

*M. tuberculosis* was reported to produce membrane-damaging proteins associated with the ESX-1 secretion system, which are required for induction of phagosomal rupture and bacterial access to the cytosolic compartment of infected phagocytic cells^4^, However, it remains unknown if other bacterial factors, as for example PLCs, might also contribute to the *M. tuberculosis*-mediated disruption of the phagosomal membrane.

The first main objective of our study was thus to investigate whether the biological activities of ESX-1 and mycobacterial PLCs were linked. For this purpose, we constructed a PLC-deletion mutant in the *M. tuberculosis* H37Rv genetic background, and subjected it to dedicated cell-biological analyses in comparison with the wild-type (WT) *M. tuberculosis* H37Rv strain. To evaluate whether the PLC-deletion mutants had the ability to induce phagosomal rupture in host-macrophages, we used a recently developed fluorescence resonance energy transfer (FRET)-based method^23^,^24^. Results from the phagosomal rupture assay together with virulence tests in cellular and small animal infection models allowed us to revisit the role of PLCs of *M. tuberculosis* in the infection process, which to our surprise was found to be only marginal. These results open new perspectives for future research to elucidate the biological role of PLCs in *M. tuberculosis* and related slow-growing mycobacteria.

## Results

### Genome analysis and deletion of the *plcABC* operon in an *M. tuberculosis* H37Rv genetic background

Analysis of *M. tuberculosis* genome data from public databases shows that most *M. tuberculosis* strains harbour four PLC encoding genes. These genes, named *plcA, plcB, plcC* and *plcD* are located at two different genomic loci in *M. tuberculosis*, with *plcA-B-C* organised as an operon (*rv2351c-rv2350c-rv2349c*) at genome coordinates 2632–2627 kb (reverse strand) of strain H37Rv, and *plcD*, represented as a single gene (*rv1755c*), located about 640 kb upstream of *plcA-C*^25^. It is also known that PLC encoding genes are preferred integration sites (or hotspots) for the IS*6110* insertion element, which may lead to the presence of two insertion elements in close proximity, favouring homologous recombination between the adjacent IS*6110* elements and deletion of the intervening sequences^26^,^27^,^28^. The widely used reference strain *M. tuberculosis* H37Rv shows such IS*6110*-mediated truncation of the *plcD* gene^26^. However, despite *plcD* inactivation, *M. tuberculosis* H37Rv retains a fully virulent phenotype in mice^29^. We thus chose the H37Rv strain to construct a null PLC mutant, taking in consideration that truncation of *plcD* facilitated the construction of the PLC complete knock-out strain, as only the PlcA-B-C operon had to be deleted.

**Figure 1:**
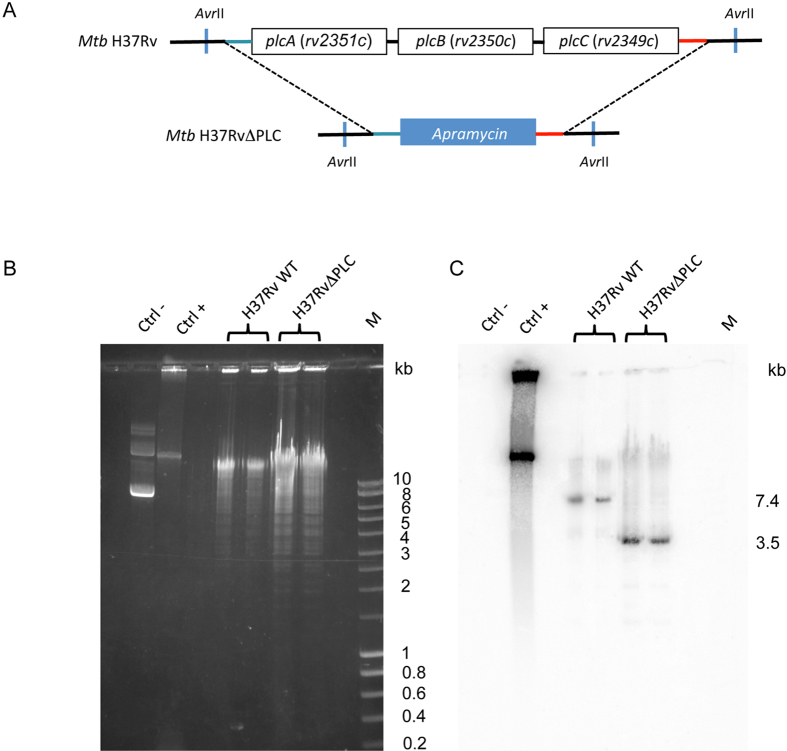
Construction of *M. tuberculosis* H37Rv*plcABC* KO (H37RvΔPLC). (**A)** Schematic representation of genomic organization of *plc* genes in *M. tuberculosis* H37Rv wild type and H37RvΔPLC strains ; (**B**) *Avr*II restriction fragment profiles of *M. tuberculosis* WT and KO strains separated by agarose gel electrophoresis; (**C**) Pattern obtained from genomic DNAs digested with *Avr*II and hybridized with a probe specific for the *plcC* downstream region; Lanes: 1 (second lane from left), negative control (pYUB412 vector); 2, positive control pYUB412::*plcABC*; 3 and 4, *M. tuberculosis* H37Rv WT, 5 and 6, *M. tuberculosis* H37Rv*ΔplcABC; 7*, M, Smart Ladder (Eurobio).

The *M. tuberculosis* H37Rv PLC null mutant (H37RvΔPLC) was constructed by using a previously described recombineering-based approach^30^. The different construction steps included the generation by 3-step-PCR^31^ of a linear DNA fragment containing an apramycin resistance cassette embedded in the flanking regions of the *plcABC* cluster (Fig. 1A), which was genetically transformed into the H37Rv strain. Selection of an appropriate clone that showed replacement of the *plcABC* cluster with the apramycin cassette was assessed by PCR and then confirmed by Southern blotting analysis (Fig. 1B,C). In addition, a H37RvΔPLC::*plcABC* complemented strain was obtained by integrating the *plcABC* gene cluster into the genome of H37RvΔPLC using the plasmid pPlcABC. This pYUB412-based vector^32^ contains the *plcABC* operon expressed under the control of its natural promoter.

As controls for selected experiments, we also included the previously described Myc2509ΔPLC mutant strain^15^, here referred to as MT103ΔPLC, and the isogenic MT103 parental *M. tuberculosis* strain.

### Evaluation of phospholipase C activity in mutant and WT *M. tuberculosis* strains

**Figure 2:**
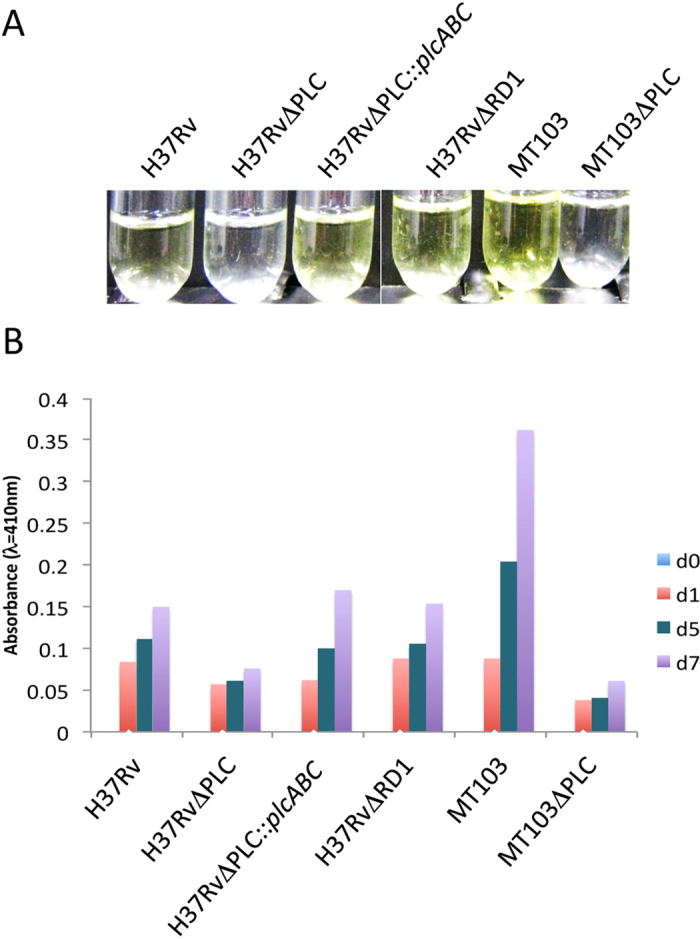
Phospholipase C enzymatic assay. This assay is based on the detection of the hydrolysis of *p-*nitrophenylphosphorylcholine (*p-*NPPC) to *p-*nitrophenol. While the substrate, *p-*NPPC, is colourless, the product p-nitrophenol due to its ability to absorb light at 410 nm, is yellow. About 500 μg of total protein were used in the assay and measurements were performed in triplicates. (**A**) Crude extracts of 4 day-old cultures from different *M. tuberculosis* strains were incubated with 5 mmol.l^−1^ of *p*-nitrophenol phosphorylcholine; (**B**) Measurement of phospholipase activity of different *M. tuberculosis* strains over 3 timepoints. Measurements were performed in duplicates.

In a first step, we used a spectrophotometric assay to determine the PLC activity of whole-cell extracts from WT *M. tuberculosis* strains and the two PLC-deletion mutants. This assay is based on the detection of the hydrolysis of colourless *p-*nitrophenylphosphorylcholine (*p-*NPPC) to *p-*nitrophenol, which absorbs light at 410 nm and is yellow. As shown in Fig. 2, the PLC activity was decreased in H37RvΔPLC compared to the corresponding WT strain. However, a lower PLC activity was detected in *M. tuberculosis* H37Rv strain relative to the MT103 strain, which might be linked to the truncation of the fourth *plc* gene (*plcD*) in *M. tuberculosis* H37Rv. Complementation of *M. tuberculosis* H37RvΔPLC with plasmid pPlcABC restored PLC activity to the level of the H37Rv WT *M. tuberculosis* strain. As expected, the *plcABC*-unrelated *M. tuberculosis* H37Rv mutant ∆ESX1, which is lacking a functional ESX-1 secretion system due to the deletion of the region of difference RD1^23^,^24^,^33^, showed a phospholipase C activity very similar to the H37Rv WT strain (Fig. 2A,B). Taken together, these results confirmed the loss of PCL activity in the H37RvΔPLC mutant.

### Phospholipase C is not involved in *M. tuberculosis*-induced phagosomal rupture

**Figure 3:**
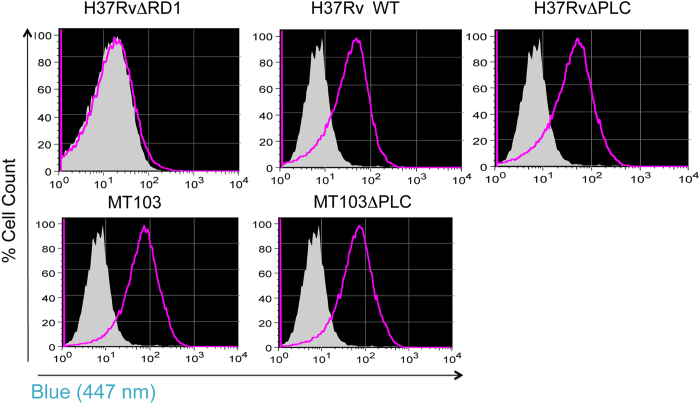
Phagosomal rupture by *M. tuberculosis* mutants. Capacity of different *M. tuberculosis* strains and mutants to induce phagosomal rupture inTHP-1 cells, monitored by CCF-4 staining and flow cytometric analysis. Non infected (gray), infected (MOI = 1:10) with different strains of *M. tuberculosis* (purple) using a recently developed approach^23^. Results shown are representative of 2 independent experiments. The shift towards blue emission (447 nm) of CCF-4 is due to the inhibition of FRET and is proportional to the mycobacteria-induced phagosomal rupture/cytosolic access.

According to host-pathogen interaction data reported from a range of bacterial pathogens, phospholipase C activity is often required for egress of bacteria from phagosomal containment and cytosolic access^20^,^21^,^22^. We thus investigated the ability of PLC mutants H37RvΔPLC and MT103ΔPLC and WT strains to access the cytosol during infection of THP-1 human macrophage-like cells, by using a recent flow-cytometric phagosomal rupture screening method^23^. Briefly, this sensitive assay relies on the change in the emission spectrum of the cephalosporin-like FRET substrate CCF-4 upon cleavage by β-lactamases^34^,^35^, which serves as a readout for the detection of contact between β-lactamase-producing *M. tuberculosis* and the FRET substrate in different environments, including the host cytosol. As CCF-4 cannot enter an intact vacuole, the assay assesses whether cytosolic contact of *M. tuberculosis* occurs in the host cell during infection. Differentiated THP-1 cells were infected with the various *M. tuberculosis* strains at an MOI of 1:2, and the CCF-4 emission spectrum of cells was monitored over a three-day period. Both the WT and the ΔPLC-deletion *M. tuberculosis* strains were able to induce a switch in the emission spectrum from ~535 nm to ~450 nm, indicating that they were gaining access to the cytosol of the infected THP-1 cells (Fig. 3). In contrast, the attenuated ΔESX-1 (ΔRD1) *M. tuberculosis* strain, which is impaired in inducing phagosomal rupture in host cells^23^,^24^ and was included in the analysis as a negative control, was unable to induce a FRET inhibition, thereby validating the assay (Fig. 3, Supplementary Figure 1). From these results we concluded that PLC of *M. tuberculosis* was not required for inducing phagosomal rupture in THP-1 cells, neither in the H37Rv-, nor in the MT103 genetic backgrounds.

### Virulence of *M. tuberculosis* H37RvΔPLC in the THP-1 infection model

**Figure 4:**
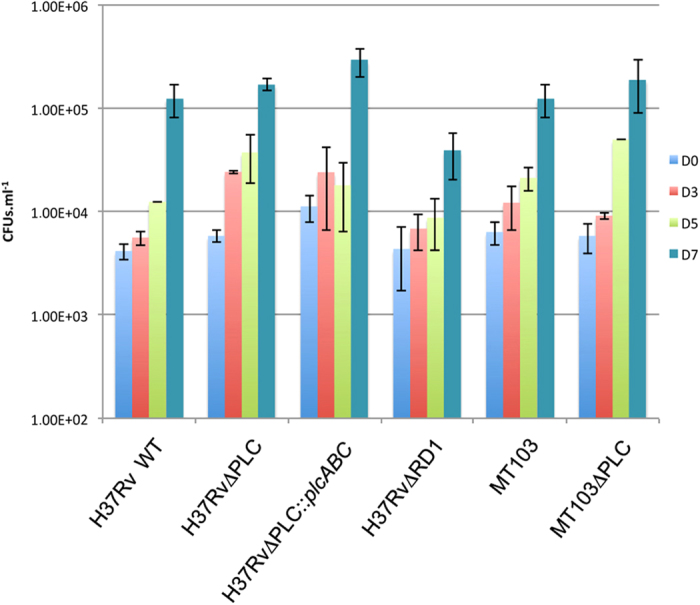
Growth kinetics of *M. tuberculosis* strains in THP-1 derived macrophages. Number of colony forming units (CFU) obtained at different time points after infection. MOI was 1:20 (bacteria/cells). The figures show the means and the standard deviations obtained in 3 independent experiments.

As phagosomal rupture in *M. tuberculosis* is often linked to virulence^36^, we evaluated the survival and/or growth of the WT and ΔPLC-mutant *M. tuberculosis* strains in THP-1 cells. WT and mutant *M. tuberculosis* strains were used to infect THP-1 cells at an MOI of 1:20 (1 bacterium per ~ 20 cells), and the number of intracellular bacteria was determined immediately after phagocytosis (day 0) and 3, 5 and 7 days post infection. As shown in Fig. 4, the H37RvΔPLC mutant and the corresponding WT strain showed similar intracellular growth kinetics, resulting in a 1.5-Log increase in CFU number over a 7-day period. Consistent with previous observations from Raynaud and colleagues^15^, no differences were observed in the intracellular growth abilities of MT103ΔPLC and its isogenic parental strain. In contrast, the ΔESX-1 (ΔRD1) *M. tuberculosis* strain, showed attenuated growth relative to WT and ΔPLC strains (Fig. 4). These results indicate that PLCs, in contrast to the ESX-1 proteins, are not essential for *M. tuberculosis* intracellular survival and optimal growth in host macrophages.

### Virulence of *M. tuberculosis* in mouse infection models

To further test whether PLC inactivation might result in a potential defect in *in vivo* growth ability that might not be detectable in macrophages cell lines, we evaluated the virulence properties of the H37RvΔPLC and WT strains in different mouse infection models.

**Figure 5:**
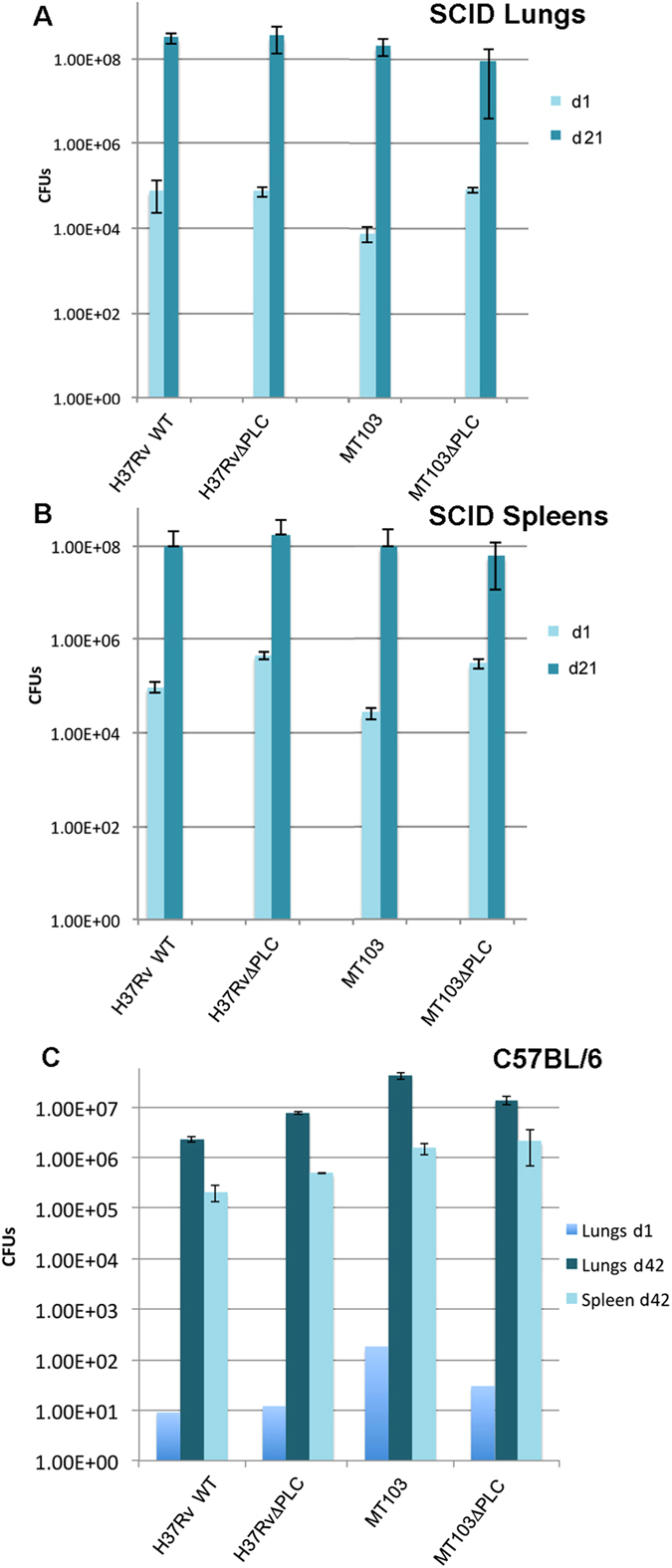
Virulence evaluation of *M. tuberculosis* strains in different mouse infection models. Number of colony forming units (CFU) 3 weeks days after intravenous infection with *M. tuberculosis* WT and mutant strains in (**A**) lungs; and (**B**) spleens of SCID mice. (**C**) Panel C shows the *in vitro* growth characteristics of the same panel of strains as above, in C57BL/6 mice 6 weeks after infection. Results shown are representative of 2 independent experiments.

Given the previously established suitability of the SCID (severe combined immune deficient) mouse infection model for distinguishing attenuated ΔESX-1 (ΔRD1) and virulent WT *M. tuberculosi*s strains^33^,^37^,^38^, the potential impact of PLC-inactivation on virulence of *M. tuberculosis* was first assessed by testing the *in vivo* growth characteristics of ΔPLC and WT *M. tuberculosis* strains in SCID mice. Both the H37Rv ΔPLC mutant and the WT strain displayed an indistinguishable, high bacterial load in lungs and spleen of infected mice (Fig. 5A,B). This was also confirmed by visual inspection of the organs, which showed typical signs of massive infection (Supplementary Figure 2). Similarly, the MT103ΔPLC and WT strains both showed comparable, intense *in vivo* growth in SCID mice, as indicated by the presence of ~10^8^ CFU in the organs after 3 weeks of infection, although it should be mentioned that for this latter strain couple, the CFU numbers determined for day 1 were somewhat higher for the ΔPLC mutant in comparison with the WT strain (Fig. 5A,B).

To determine whether the findings obtained in the SCID mouse model were also relevant in immunocompetent mice, virulence studies with the H37Rv and MT103 ΔPLC and WT strain-pairs were also performed in an aerosol infection model of C57BL/6 mice, where the bacterial load in target organs was determined after 6 weeks of infection. As shown in Fig. 5C and Supplementary Figure 3, we did not observe a significant difference between WT and ΔPLC mutants in their *in vivo* growth properties. These findings, which are in overall agreement with results from the phagosomal rupture screen and the THP-1 infection assay, suggest that PLCs from *M. tuberculosis* might not represent very obvious virulence factors of *M. tuberculosis*.

### Expression of *plcABC* genes seems to linked to phosphate concentration

**Figure 6:**
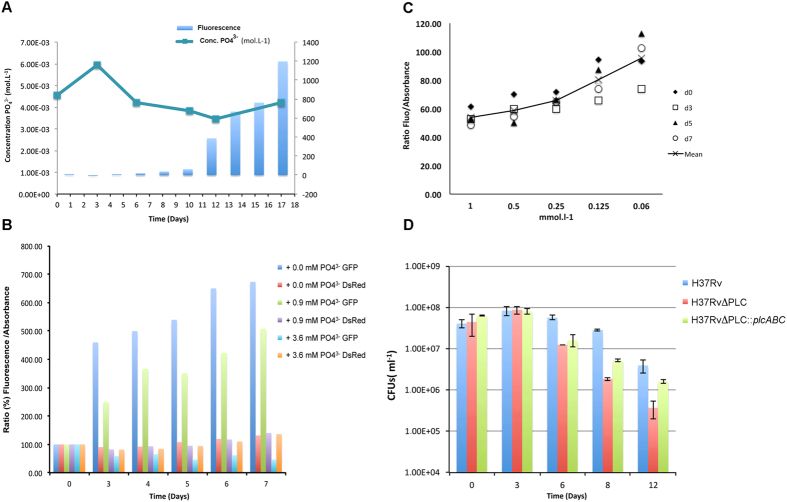
*plcA-egfp* fusion gene expression of *M. tuberculosis* H37RvΔPLC::Pr_*plcA-egfp* during growth in phosphate limiting conditions. (**A**) The curve shows the phosphate concentration in samples over time of *in vitro* growth. Histogram represents increase of the culture fluorescence intensity due to GFP expression over time. Results shown are representative of 2 independent experiments. Note that towards the end of the experiment the phosphate concentration slightly increased, which is plausibly due to lysis of some of the older bacterial cells. (**B**) Measurement of fluorescence divided into GFP and red fluorescence in a culture of H37RvΔPLC::Pr_*plcA*-*egfp* complemented with a DsRed expressing plasmid. DS-red is expressed via a constitutive promoter while GFP expression is dependent on *plcA* promoter activity. (**C**) Promoter activity of *M. tuberculosis* H37RvΔPLC::Pr_*plcA*-*egfp* in presence of decreasing phosphate concentration due to *in vitro* growth of culture during 7 days at 37 °C under shaking conditions. Measures show the ratio between fluorescence and absorbance, the first reflecting GFP expression levels and the latter reflecting cell density. (**D**) Survival of *M. tuberculosis* H37Rv WT and mutant strains in broth that provides phosphatidylcholine as the sole phosphate source. Results shown represent two different experiments. For each experiment the different strains tested were plated and counted in triplicate.

Previous studies on PLCs of *P. aeruginosa* have shown that induction of PLC expression was phosphate regulated, suggesting a putative role of PLCs for retrieval of phosphate from the environment^20^,^39^. To investigate whether PLCs of *M. tuberculosis* might present similar features, we established an *in vitro* growth model under phosphate-limiting conditions (Supplementary Figure 4). For monitoring promoter activities, a recombinant ΔPLC *M. tuberculosis* H37Rv strain expressing a translational 5′-*plcA*-*egfp* fusion under the natural *plcABC* promoter was constructed and named H37RvΔPLC::*plcA*-*egfp.* Results obtained from growth experiments with this strain showed that during the first 9 days fluorescence remained low, while starting from day 10 post-inoculation a strong increase in fluorescence was noted (Fig. 6A). By this time-point the phosphate concentration in the medium was below 0.3 mmol.l^–1^. The expression of the *plcABC* genes thus seems to be induced by low phosphate concentration, although an impact of other potential stress factors linked to the consumption and limitation of essential nutriments may not be excluded. To further investigate this point, an H37RvΔPLC::*plcA*-*egfp* strain that also expressed DsRed under a constitutive promoter was constructed. With the help of this strain promoter activity was studied at different phosphate ion concentrations, simultaneously controlling for the impact of cell density on fluorescence levels. Monitoring of green fluorescence relative to red fluorescence and absorbance levels showed that under low phosphate conditions green fluorescence increased strongly relative to the constant level of red fluorescence, confirming that the *plcABC* promoter was more strongly induced under low phosphate conditions (Fig. 6B). Finally, we also evaluated the GFP-fluorescence normalized to the cell density measured in OD, and again observed that at low phosphate concentration the promoter activity of the *plcABC* operon was increased (Fig. 6C). Starvation of phosphate ions thus seems to represent a stress that the bacteria try to counterbalance by induction of PLC production.

Finally, we also conducted experiments wherein the WT, ΔPLC and ΔPLC::*plcABC* H37Rv *M. tuberculosis* strains were grown in liquid medium supplemented with phosphatidylcholine as the sole phosphate source. As shown in Fig. 6D, the WT *M. tuberculosis* H37Rv strain and the complemented strain survived better under these conditions compared to the ΔPLC mutant. It should be emphasized, however, that none of the strains was able to actively grow under these experimental settings.

## Discussion

PLCs are widely distributed enzymes in living organisms. PLCs hydrolyze phospholipids such as phosphatidylcholine or sphingomyelin at the phosphodiester bond. In bacteria, these enzymes have been reported to function in a wide variety of cellular tasks during infection, including membrane lysis, intracellular signalling, lipid metabolism and/or pathogenicity-associated activity^40^,^41^. In our study, we focused on the PLCs of *M. tuberculosis*, which belong to the superfamily of haemolytic phosphocholine-specific PLCs for which PLC of *P. aeruginosa* is the paradigm member^42^. Our initial objective was to evaluate whether these enzymes were involved in the process of phagosomal rupture induced by *M. tuberculosis* during infection of macrophages. In *L. monocytogenes*, or *C. perfringens*, PLCs play important roles together with pore forming listeriolysin or perfringolysin, respectively, to lyse the phagosomal membrane and allow the bacteria to gain access to the cytosol and promote cell-to-cell spread^22^,^43^. Concerning the infection with *M. tuberculosis*, the scenario seems more complex. While it was long thought that *M. tuberculosis* resists degradation in the phagosome by inhibiting the fusion with lysosomes, favoring intra-phagosomal survival and multiplication^44^, more recent studies by van der Wel and colleagues, using cryo-electron microscopy, provided evidence of cytosolic presence of *M. tuberculosis* at later stages of infection^45^. Similarly, cytosolic access of virulent *M. tuberculosis* strains was recently also reported by using a FRET-based read-out, combined with automated confocal microscopy^24^ or flow cytometry^23^. We here used the latter method to test the *M. tuberculosis* ΔPLC and WT strains for their ability to cause phagosomal rupture in comparison with a ΔESX-1 (ΔRD1) negative control and found that the *M. tuberculosis* ΔPLC mutants and WT strains all showed very similar abilities to gain cytosolic access.

Given the result that PLCs of *M. tuberculosis* were not required for inducing phagosomal rupture and cytosolic contact of *M. tuberculosis*, which are attributes usually linked to mycobacterial pathogenicity^36^, we subjected the ΔPLC mutants and WT strains to virulence analyses in *in vitro/ex vivo* and *in vivo* models. In the obtained data, we could only detect minor, not significant virulence differences between the ΔPLC and WT *M. tuberculosis* strains of two genetic backgrounds, i.e. MT103 and H37Rv, in the 3 models used. These results, which were different from those of previous work reporting that PLCs were involved in virulence of *M. tuberculosis*^15^, remained puzzling.

Review of the available literature suggests that the number of functional PLC-encoding genes in different strains of the *M. tuberculosis* complex is highly variable and ranges from 0 to 4 copies. In many *M. tuberculosis* strains, including H37Rv, the *plcD* gene, which represents a hotspot for IS*6110* insertion, is inactivated or deleted^27^,^28^,^46^. Similarly, extensive IS*6110* insertion is also observed for the *plcABC* locus, but to a lesser extent^47^. Moreover, in a study on genetic polymorphisms affecting the four PLC encoding genes in *M. tuberculosis* isolates, Viana-Niero and coworkers found that 19 of 25 clinical isolates showed loss of parts of genes or complete genes from the *plcABC* and/or *plcD* loci, whereby five isolates retrieved from patients with active tuberculosis had all 4 plc genes interrupted^48^. PLC-encoding loci are also variable in different lineages of the *M. tuberculosis* complex; PlcA/B/C, which are also known as the “mtp40” mycobacterial protein(s), are missing from the *M. bovis* lineage due to the RD5 deletion, and also are absent from certain other tubercle bacilli^27^,^49^. In this respect it is also noteworthy that *M. bovis* strains with an IS*6110* insertion in the remaining *plcD* gene were described. Interestingly, these strains without a functional PLC encoding gene were responsible for causing tuberculosis lesions in cattle for which no differences in the organ distribution relative to other *M. bovis* strains were noticed^50^. These findings are also in agreement with results from a high-density transposon screen, wherein PLC-encoding genes have not been identified as essential for *in vivo* growth of *M. tuberculosis* in the mouse model^51^. Taken together, these reports and our experimental findings with two different ΔPLC mutants of *M. tuberculosis* cast doubt on an essential role of PLC in virulence of tubercle bacilli. PLCs of *M. tuberculosis* might play a less important role in the infectious lifecycle of *M. tuberculosis* than previously thought.

However, it is intriguing that despite the apparently marginal role of PLCs in virulence of *M. tuberculosis*, most strains have conserved one or more copies of PLC-encoding genes in their genomes, similar to certain non-tuberculous (NTM) mycobacteria. There are only few mycobacterial species that harbour genes encoding PLCs. Database analyses show that for the group of slow-growing mycobacteria, PLC-encoding genes are present in the genomes of smooth tubercle bacilli^52^, members of the *M. tuberculosis* complex, members of the *Mycobacterium kansasii*-*Mycobacterium gastri* cluster, *Mycobacterium asiaticum*, and members of the *Mycobacterium marinum*-*Mycobacterium ulcerans* cluster. PLCs are absent from the genomes of *Mycobacterium leprae*, and members of the *Mycobacterium avium-intercellulare* complex. In the more distantly related rapid growing mycobacteria, only *M. abscessus* is known to carry a PLC, which shows 37% amino-acid identity with PLCs from *M. tuberculosis* and seems to be the result of a specific horizontal gene transfer (HGT) into *M. abscessus*^13^,^53^. In contrast, the PLCs in *M. tuberculosis* and other slow-growing mycobacteria seem to share a common origin with PLCs from different *Gordonia* species, with which they show about 60% amino acid identity. It seems thus likely that a common progenitor of the phylogenetic subgroup of slow-growing mycobacteria comprising *M. kansasii*, *M. gastri*, *M. asiaticum*, *M. marinum* and *M. tuberculosis* has acquired the PLC-encoding genes during evolution through HGT from more distantly related actinobacteria.

This feature prompted us to search for potential alternative biological functions of PLCs in slow growing mycobacteria, not necessarily linked with virulence. As one hypothesis, PLCs of *M. tuberculosis* might help in the acquisition of phosphate. Cleavage of phospholipids by PLCs results in the generation of two molecular entities, a glycerol part and a residue containing a phosphate group, which might serve as a potential source of phosphate for the bacterium. The finding that expression of the *plcA-egfp* fusion was inversely correlated with the phosphate concentration in the medium suggests that the specific promoter activity might be downregulated under phosphate-sufficient environmental conditions. This assumption is in agreement with previous observations with PLCs from *P. aeruginosa*, for which an impact of phosphate concentration on *plc* gene regulation was noted^20^,^39^. Moreover, it has previously been reported that during infection of THP-1 cells by *M. tuberculosis*, the expression of the *plcABC* operon was upregulated for the first 24 h of infection^15^. Given the results obtained with our GFP-fusion assay, it is thus tempting to speculate that this upregulation might be related to a limited phosphate concentration inside the phagosome. Limitation of phosphate during phagosomal containment was also postulated by results from large-scale transcriptome studies, which found genes encoding phosphate transporters upregulated during infection^18^,^54^. It is plausible that *M. tuberculosis* can vary its supply in phosphate between inorganic phosphate, which is the preferred source of phosphorus for many bacteria^18^, and acquisition of organic phosphates through the action of phosphatases and/or phospolipases. A similar scenario was recently suggested for SpmT (Rv0888) of *M. tuberculosis*, which harbours a surface-exposed C-terminal sphingomyelinase domain and a putative N-terminal channel domain that mediates glucose and phosphocholine uptake across the outer membrane^55^. However, at present it remains unknown if the PLCs of *M. tuberculosis* may contribute to the phosphate supply of the bacterium in a similar way. Our results point to such a possibility, although more in depth studies are needed to clarify this question.

In conclusion, our study calls into question the impact of PLCs on virulence of *M. tuberculosis*, and provides new hints on putative alternative functions of PLCs in *M. tuberculosis*.

## Methods

### Bacterial strains and culture conditions

*Escherichia coli* DH10B and Top10 (Invitrogen) strains, used for cloning procedures, were grown on LB agar medium and/or LB broth. *M. smegmatis* mc^2^155 and *M. tuberculosis* strains were obtained from stock held at the Institut Pasteur. The *M. tuberculosis* MT103 strain and the corresponding Myc2509ΔPLC mutant strain^15^ were a gift of Prof. Gicquel, Institut Pasteur.

Mycobacterial strains were cultured in Middlebrook 7H9 broth supplemented with ADC (Difco) and 0.05% Tween 80 or on Middlebrook 7H11 medium supplemented with OADC (Difco). When required, antibiotics were included for selection purposes at following concentrations: Hygromycin (200 μg.ml^–1^) and Zeocin (25 μg.ml^–1^) for *E. coli;* Hygromycin (50 μg.ml^–1^), Apramycin (50 μg.ml^–1^), Kanamycin (25 μg.ml^–1^), Zeocin (25 μg.ml^–1^), for mycobacteria.

For the PlcABC-promoter induction assay, phosphate-free Sauton medium was prepared as follows: L-asparagine 4 g/L, MgSO_4_-7 H_2_O 0.5 g/L; ammonium iron III citrate 0.05 g/L; citric acid 2 g/L; ZnSO_4_ 1% 0.1 ml/L; glycerol 60 ml/L. pH was adjusted between 7.2–7.3 by a buffer solution of ammonium hydroxide.

### Construction of a *plcABC* deletion mutant in *M. tuberculosis* H37Rv

The *M. tuberculosis* H37Rv *ΔplcABC* mutant was constructed by allelic replacement using the recombineering method^30^. The allelic exchange substrate *plcABC*::Apra was obtained by a three step PCR approach^56^. Briefly, two 500–bp fragments corresponding to the *plcABC* upstream and downstream regions were amplified by PCR from the *M. tuberculosis* H37Rv genomic DNA and linked to a third PCR fragment encoding the apramycin resistance cassette, to generate the 2 kb-fragment *plcABC*::Apra. The *plcABC*::Apra fragment was thus used to transform a *M. tuberculosis* H37Rv recombinant strain containing the pJV53 vector. The pJV53 plasmid encodes the recombination proteins gp60 and gp61^57^, whose expression is induced by incubation with^30^ 0.2% acetamide for 24 h. The H37Rv*-*pJV53 acetamide-activated transformants were selected on solid medium for resistance to Kanamycin and Apramycin. The obtained Kanamycin and Apramycin resistant clones were thus tested for the plcABC deletion by PCR. One out of 116 tested clones revealed an amplification profile consistent with the replacement of the *plcABC* cluster with the apramycin cassette, and was thus subjected to Southern blot analyses. Genomic DNAs from *M. tuberculosis* strains were digested with *Avr*II, separated by gel electrophoresis and transferred onto Hybond-C-Extra nitrocellulose (GE). Hybridization was performed with [α-32 P] dCTP-labeled PCR-probe, specific for the *plcABC* downstream region, in 6x SSC, 0.5% SDS, 0.01 M EDTA, 5x Denhardt’s solution, 100 μg.ml^−1^ salmon-sperm DNA, at 68 °C. After washing, membranes were exposed to phosphorimager screens, which were scanned in a STORM phosphorimager^31^,^57^.

### Construction of *M. tuberculosis* H37RvΔPLC complemented strains

Two different integrative pYUB412-based plasmids (pPlcABC and pYUB412-Pr_*plcA*-*egfp*) harbouring the *plcABC* operon and the plcA gene, respectively, were constructed. To obtain the pPlcABC plasmid, the *plcABC* operon and its natural promoter region, were amplified by PCR and cloned into the pYUB412 vector backbone. Similarly, to construct the pYUB412-Pr_*plcA*-*egfp* plasmid, the *plcA* gene and the *plcABC* promoter region were amplified by PCR using modified primers (Supplementary Table 1), which allow the introduction of additional *Hin*dIII and *Nhe*I recognition sequences in the amplified fragment obtained. The resulting PCR product was digested and ligated into the *Hin*dIII-*Nhe*I-digested pYUB412::*egfp* (a pYUB412 derivative cosmid that allows the expression of transcriptional eGFP fusion protein constructs^32^).

Both pPlcABC and pYUB412-Pr_*plcA*-*egfp* constructs were used to transform the *M. tuberculosis* H37RvΔPLC mutant strain. Transformed clones were selected on solid medium for resistance to hygromycin.

### PCR amplification and DNA Sequencing

PCR reactions to obtain fragments used in cloning procedures or in screening of transformed clones were carried out with Pwo (Roche) or similar high fidelity DNA polymerases, respectively, as previously reported^58^. Sequences of primers used in amplification reactions are listed in Table S1. All amplified PCR products and plasmids were sequenced by using the Big Dye cycle sequencing Kit (Applied Biosystems) in an automated DNA sequencer (Applied Biosystems, 3130xl genetic analyser).

### Transformation of mycobacterial strains

To obtain mycobacterial competent cells, *M. tuberculosis* H37Rv and *M. smegmatis* Mc^2^155 were recovered from cultures at exponential growth, and washed three times in 10% glycerol. Aliquots (100 μl) of freshly prepared electro-competent cells in glycerol 10% were transformed with 100 - 200 ng of vector DNA in 0.2-cm cuvettes (2.0 kV; 25 μF : 1000 Ohms) at room temperature^58^. Transformant clones were selected by incubation on solid medium supplemented with the corresponding antibiotic for 2–3 weeks at 37 °C.

### Phospholipase C assay

PLC activities were measured using the *p-*nitrophenylphosphorylcholine (*p*-NPPC) substrate (Sigma-Aldrich). Activity is defined as the ability of an enzyme to catalyse *p*-NPPC into *p-*nitrophenyl (*p-*NP) of yellow chromogenic nature. Briefly, 0.5 mg of total protein was incubated in 3 ml of 10 mM Tris HCl (pH = 7.2), containing 5 mM of *p-*NPPC and 1.5% of sorbitol^16^. The reaction mix was incubated at 37 °C under shaking at 100 rpm. The reaction was stopped after 0, 1, 2, 3, 4, or 7 days by NaOH at 0.1 N (final concentration). The release of *p-*NP was measured at 410 nm. Buffer without proteins served as blank reference. For initial experimental setup, a control assay was performed with 40 U of purified PLC from *B. cereus* (Invitrogen).

### Phosphate assay by colorimetric method

Phosphate ions in presence of L(+) ascorbic acid (Merck) and ammonium molybdate tetra-hydrate (Sigma) form a complex showing a blue/green colour. Briefly, comparator samples containing between a standard range of 0 and 6.10^−5^ mol. L^−1^ of phosphate (KH_2_PO_4_) were prepared in 15 mL glass tubes. After adding 1 mL of stock solution of ascorbic acid at 0.1 mol.L^−1^ and ammonium molybdate tetra-hydrate at 0.2 mol.L^−1^, the react volume was adjusted to 10 ml final volume with Milli-Q (Millipore) purified water. All tubes were incubated at 80 °C in a water bath during 10 minutes and slowly cooled down to room temperature on the bench. Then all samples and comparator samples were diluted (d = 1/2) before measurement at 750 nm.

### Infection of THP-1 derived macrophages with *M. tuberculosis*

THP-1 cells were grown at 37 °C with 5% CO_2_. Cells were maintained in RPMI 1640 + glutamax (Life technologies) and 10% of foetal bovine serum. THP-1 cells were seeded in 96 well plates at 7.5 × 10^4^ cells per well, and differentiated by incubation with 10 ng.ml^−1^ of PMA for 2 days. Before infection, the medium was removed and the wells were washed 3 times with PBS. Bacterial strains were added at a multiplicity of infection (MOI) = 1: 20 (1 bacterium: 20 macrophages). After 2 hours (day 0) or 3, 5 and 7 days post-infection, cells were lysed in PBS 0,01% of Triton X-100. The number of viable intracellular mycobacteria was determined/counted by plating serial dilutions of macrophage lysates on solid medium.

### Phagosomal rupture assay by flow cytometry analysis

Differentiated THP-1 cells were infected at MOI of 0.5 and were stained at day 3 post-infection with 8 μM CCF-4 (Invitrogen) in EM buffer (120 mM NaCl, 7 mM KCl, 1.8 mM CaCl2, 0.8 mM MgCl2, 5 mM glucose and 25 mM Hepes, pH 7.3) complemented with 2.5 μM probenecid, during 1 h at room temperature. Cells were then washed once in PBS and stained with anti-CD11b-APC (BD) monoclonal antibody in PBS containing 3% fetal calf serum and 0.1% NaN3 and fixed with 4% paraformaldehyde overnight at 4 °C. Cells were analyzed in a CyAn cytometer by use of Summit software (Beckman Coulter, France). Data were analyzed with FlowJo software (Treestar, OR).

### *M. tuberculosis* virulence studies in mice

Six-week-old female CB17/Ico SCID mice (Charles River) were infected intravenously via the lateral tail vein with 200 μl of bacterial suspension of 1 × 10^6^ CFU.ml^−1^. For aerosol infection, a customized apparatus was used following a previously established procedure^59^ Six-week-old female C57BL/6B mice (Charles-River) were infected with a suspension containing 5 × 10^5^ bacteria.ml^−1^ to obtain an inhaled dose of ca. 100 CFU. At selected time points after infection, mice were killed and organs homogenised using a tissue Lyser apparatus from Quiagen and 2.5 mm diameter glass beads to determine CFU numbers as previously reported^52^,^59^.

All animal studies were approved by the Institut Pasteur Safety Committee (Protocol 11.245; experimentation authorization number 75–1469), in accordance with European and French guidelines (Directive 86/609/CEE and Decree 87–848 of 19 October 1987), and implicating approval from local ethical committees (CETEA 2013–0036).

### *plcABC*-promoter induction assay

The H37RvΔPLC::Pr_plcA-*egfp* strain was complemented with a DsRed expressing plasmid. The resulting strain was named H37RvΔPLC::Pr_plcA-*egfp*::Pr-hsp60*-*DsRed. In this construct DsRed is expressed *via* a constitutive promoter while GFP expression is dependent on *plcA* promoter activity. Briefly, for these experiments bacteria were grown in 7H9 + ADC + Hygromycin 50 μg.ml^−1^/Zeocin 25 μg.ml^−1^ medium until 0.4–0.6 OD. Bacteria of this preculture were inoculated in fresh Sauton medium containing 0.05% Tween80 and Hygromycin 50 μg.ml^−1^/Zeocin 25 μg.ml^−1^ at 0.05 OD. After 7 days of culture, bacteria were harvested and centrifuged during 5 min at 5000 g. After 3 washing steps, using 5 ml of fresh Sauton medium lacking PO_4_^3–^, bacteria were inoculated at 0.05 final OD in Sauton medium containing phosphate or not. In addition, the bacterial quantities were monitored by plating aliquots of each strain onto agar plates (in triplicate) for CFU determination. The GFP (Ex = 475 nm/Em = 504 nm) and DsRed fluorescence (Ex = 558/Em = 583) was monitored using a microplate reader (BGM Labtech) and analysed by Omega software. To avoid an increase of fluorescence due to the growth, we chose to normalize fluorescence values relative to absorbance. These ratio measurements were converted in percentage, with the day 0, as reference point.

### Mycobacterial phosphatidylcholine survival assay

*M. tuberculosis* H37Rv WT, *ΔplcABC* and complement strains were inoculated into phosphate-free Sauton medium supplemented with phosphatidylcholine (3.6 mM) (Sigma) as the sole phosphate source, considering that 1 mole of phosphate was equal to 1 mole of phosphatidylcholine. Addition of phosphatidylcholine rendered the medium turbid, which precluded the use of OD measurement. CFU counting was used as an alternative for quantification of bacteria, as previously reported^60^.

### Statistical analyses

Potential statistical differences in bacterial loads were evaluated by ANOVA test with Tukey correction, after conversion of CFU numbers in Log10 CFU values. Statistical significance was considered to be a P value ≤ 0.05.

## Additional Information

**How to cite this article**: Le Chevalier F. *et al.* Revisiting the role of phospholipases C in virulence and the lifecycle of *Mycobacterium tuberculosis*. *Sci. Rep.* **5**, 16918; doi: 10.1038/srep16918 (2015).

## Supplementary Material

Supplementary Materials
