# Supplementary material for: Revisiting the role of phospholipases C in virulence and the lifecycle of Mycobacterium tuberculosis
Source: Sci Rep. 2015 Nov 25;5:16918. doi: 10.1038/srep16918 (PMC4658479; doi:10.1038/srep16918)
Supplement: Supplementary Materials [file srep16918-s1.pdf]

Supplementary Information:

Revisiting the role of phospholipases C in virulence and the lifecycle of *Mycobacterium tuberculosis*

Fabien Le Chevalier, Alessandro Cascioferro, Wafa Frigui, Alexandre Pawlik, Eva C. Boritsch, Daria Bottai, Laleh Majlessi, Jean Louis Herrmann, and Roland Brosch

Supplementary Figure 1: Capacity of different mycobacterial strains at incuding phagosomal rupture (Gating Strategy)

(after Simeone et al., PLoS Pathogens, 2015)

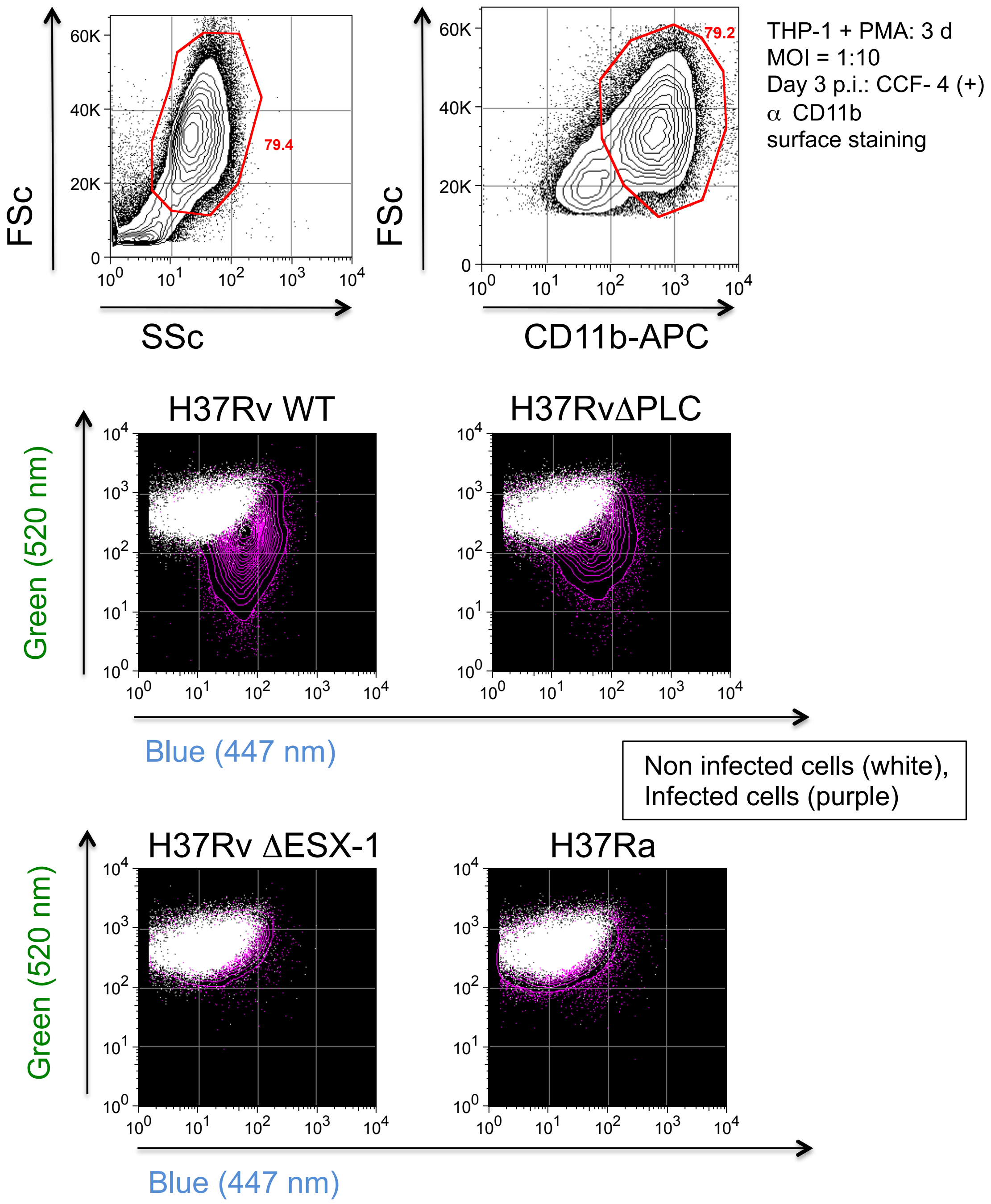

M. tuberculosis
H37Rv WT

M. tuberculosis
MT103 WT

M. tuberculosis
H37RvΔPLC

M. tuberculosis
MT103ΔPLC

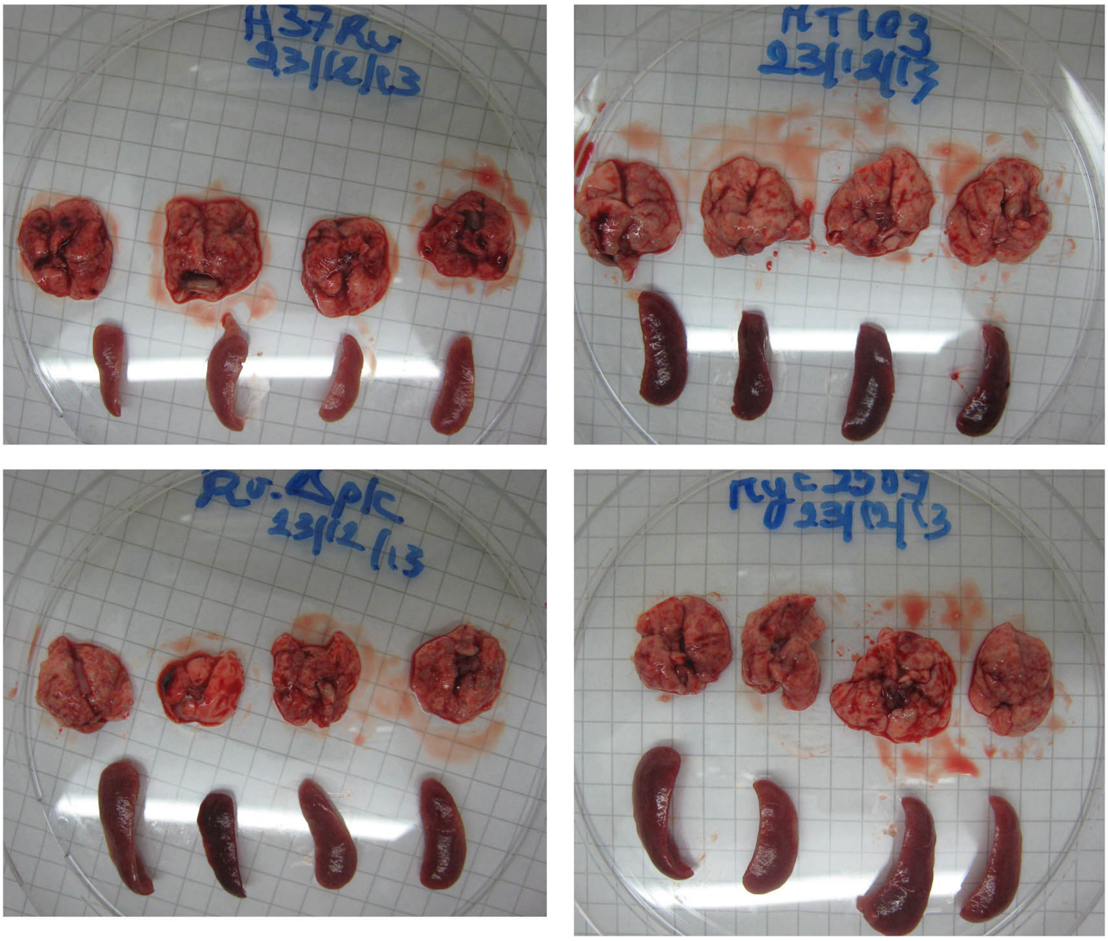

Supplementary Figure 2: Lungs and spleens from SCID mice 3 weeks after intraveneous infection with the different *M. tuberculosis* strains, indicated.

M. tuberculosis
H37Rv WT

M. tuberculosis
MT103 WT

M. tuberculosis
H37RvΔPLC

M. tuberculosis
MT103ΔPLC

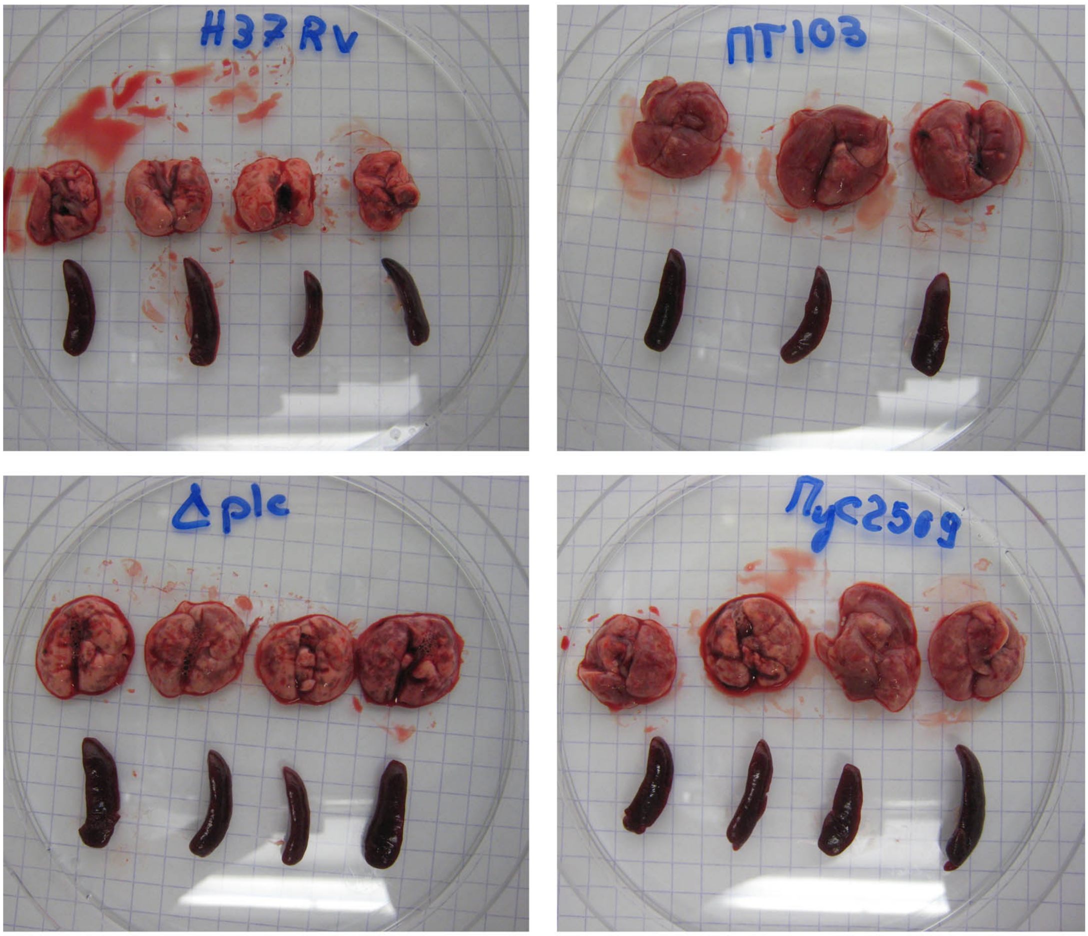

Supplementary Figure 3: Lungs and spleens from C57BL/6 mice 6 weeks after aerosol infection with the different *M. tuberculosis* strains, indicated.

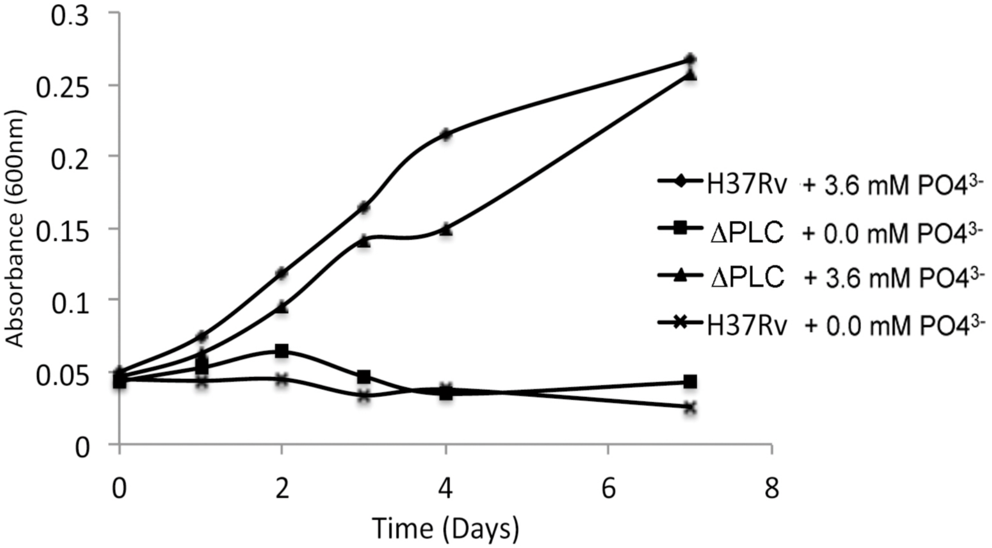

Supplementary Figure 4: Growth curve at 37°C of *M. tuberculosis* H37Rv WT and ΔPLC strains in presence or absence of phosphate.

Supplementary Table 1

Table n°1 Pimers list used in this study

	Name	Séquence
	Construction of M.tuberculosis H37RvΔ*plc*	
**1**	Apra_SwaI_Forward	5'-ATTTAAATttcatgtgcagctccatcagc-3'
**2**	Apra_SwaI_Reverse	5'-ATTTAAATcattgagcgtcagcatatcat-3'
**3**	Up-region_plcA_Del_Forward	5'-gaatgcaagcaatgggcggac-3'
**4**	Up-region_plc_Del_Reverse	5'-tggagctgcacatgaaATTTAAATtccgaggagtgggcttgcgct-3'
**5**	Up-region_plcC_Del_Forward	5'-atgctgacgctcaatgATTTAAATccggggatggttcagacgtaa-3'
**6**	Up-region_plcC_Del_Reverse	5'-atgcggaatcacgctgctgct-3'
	Screening of M.tuberculosis H37RvΔ*plc*	
**7**	Apra_Verif_Down	5'-tcgctgatgatatgctgacg-3'
**8**	Apra_Verif_Up	5'-ctgatggagctgcacatgaa-3'
**9**	Verif_H37Rv_Del_Up	5'-ccgctggccatgcggaaag-3'
**10**	Verif_H37Rv_Del_Down	5'-tcataaaacgtgttgccatgttg-3'
	Construction of Pr_plc-ATG*egfp* plasmid	
**11**	PlcA_NheI_R	5'-atcgGCTAGCtgacattccgaggagtgggc-3'
**12**	Pr_plcA_HindIII_EcoRV_F	5'-atcgAAGCTTGATATCgaatgcaagcaatgggcgga-3'
